# Carbon monoxide-releasing molecule-3 protects against ischemic stroke by suppressing neuroinflammation and alleviating blood-brain barrier disruption

**DOI:** 10.1186/s12974-018-1226-1

**Published:** 2018-06-21

**Authors:** Jianping Wang, Di Zhang, Xiaojie Fu, Lie Yu, Zhengfang Lu, Yufeng Gao, Xianliang Liu, Jiang Man, Sijia Li, Nan Li, Xuemei Chen, Michael Hong, Qingwu Yang, Jian Wang

**Affiliations:** 1 Department of Neurology, The First Affiliated Hospital of Zhengzhou University, Zhengzhou, 450052 Henan China; 2 Department of Neurology, The Second Affiliated Hospital of Zhengzhou University, Zhengzhou, 450014 Henan China; 3 Department of Anesthesiology/Critical Care Medicine, Johns Hopkins University, School of Medicine, Baltimore, 21205 MD USA; 4 Department of Anatomy, College of Basic Medical Sciences, Zhengzhou University, Zhengzhou, 450000 Henan China; 5 Department of Neurology, Xinqiao Hospital, Third Military Medical University, Chongqing, 400037 China

**Keywords:** Carbon monoxide-releasing molecule-3, Cerebral ischemia, Blood-brain barrier, Neuroinflammation, Carbon monoxide

## Abstract

**Background:**

At low levels, carbon monoxide (CO) has been shown to have beneficial effects on multiple organs and tissues through its potential anti-inflammatory, anti-apoptotic, and anti-proliferative properties. However, the effect of CO-releasing molecule (CORM)-3, a water-soluble CORM, on ischemic stroke and its mechanism of action are still unclear.

**Methods:**

We investigated the role of CORM-3 in the mouse model of transient middle cerebral artery occlusion (tMCAO). CORM-3 or saline was administered to mice by retro-orbital injection at the time of reperfusion after 1-h tMCAO or at 1 h after sham surgery. We assessed infarct volume and brain water content at 24 and 72 h after ischemia, blood-brain barrier permeability at 6 and 72 h after ischemia, and neurologic deficits on days 1, 3, 7, and 14.

**Results:**

Among mice that underwent tMCAO, those that received CORM-3 had significantly smaller infarct volume and greater expression of neuronal nuclear antigen (NeuN) and microtubule-associated protein 2 than did saline-treated mice. CORM-3-treated mice had significantly fewer activated microglia in the peri-infarction zone than did control mice and exhibited downregulated expression of ionized calcium-binding adapter molecule (Iba)-1, tumor necrosis factor-α, and interleukin 1β. CORM-3-treated mice had significantly lower brain water content and enhanced neurologic outcomes on days 3, 7, and 14 post-tMCAO. Lastly, CORM-3 treatment reduced Evans blue leakage; increased expression of platelet-derived growth factor receptor-β, tight junction protein ZO-1, and matrix protein laminin; and decreased protein level of matrix metalloproteinase-9.

**Conclusion:**

CORM-3 treatment at the time of reperfusion reduces ischemia-reperfusion-induced brain injury by suppressing neuroinflammation and alleviating blood-brain barrier disruption. Our data suggest that CORM-3 may provide an effective therapy for ischemic stroke.

## Background

Ischemic stroke is the most common form of stroke and a major cause of disability and death in adults worldwide. The obstruction of cerebral blood flow initiates the acute phase of cerebral injury, including neuroinflammation and cerebral edema [1]. Neuroinflammation is a crucial contributor to neuronal death after ischemic stroke, and activated microglia have been the object of studies concentrating on neuroinflammation [2–5]. Cerebral edema, mainly resulting from the increase of cerebral vascular permeability, is the most common cause of neurologic deficiency and mortality during acute ischemic stroke, highlighting the importance of maintaining blood-brain barrier (BBB) integrity [6–9]. Despite enormous efforts in both preclinical and clinical research, treatments to protect against ischemic stroke are still insufficient.

A novel approach to protecting against ischemic stroke injury is the administration of low levels of exogenous CO [10]. Endogenous carbon monoxide (CO) is a product of heme catabolism by heme oxygenase (HO) [11–14]. Exogenous CO is commonly regarded as poisonous because its high affinity for hemoglobin can cause rapid elevation of carboxyhemoglobin (COHb) to toxic levels that compromise oxygen delivery to the tissues [11]. However, accumulating evidence suggests that low levels can offer protection through potential anti-inflammatory, anti-apoptotic, and anti-proliferative effects [15–17]. Carbon monoxide-releasing molecules (CORMs), a group of compounds capable of carrying and liberating controlled quantities of CO, have shown promise for delivering exogenous CO without altering COHb to toxic levels [11, 18–20]. Although the mechanisms by which CORM-derived CO might offer its beneficial effects have not yet been thoroughly investigated, many studies substantiate the protective role of CORM-derived CO against cellular and tissue damage in numerous models of injury, such as renal ischemia-reperfusion injury, hemorrhagic stroke, traumatic brain injury, transplantation, sepsis, hypertension, and cardiovascular disorders [21–28].

Currently, the effect of water-soluble CORM-3 [11, 22, 24] on ischemic stroke remains unclear. In this study, we investigated the therapeutic potential of CORM-3 for ischemic stroke using a mouse model of transient middle cerebral artery occlusion (tMCAO). We found that CORM-3 treatment may contribute to better outcomes after cerebral injury by attenuating brain infarct and edema, lessening BBB disruption, and improving neurologic functions. Further investigation into mechanisms underlying the beneficial effects of CORM-3 on ischemic stroke revealed that CORM-3 increased the protein levels of neuronal nuclear antigen (NeuN) and microtubule-associated protein 2 (MAP2), and suppressed microglial activation, which reduced the quantities of reactive microglia and downregulated the expression of ionized calcium-binding adapter molecule (Iba)-1, tumor necrosis factor (TNF)-α, and interleukin (IL) 1β. CORM-3 also elevated the expression of the pericyte marker platelet-derived growth factor receptor (PDGFR)-β, tight junction protein ZO-1, and matrix protein laminin, which reduced BBB leakage. Finally, CORM-3 reduced the protein level of matrix metalloproteinase (MMP)-9. Taken together, our findings demonstrate clear benefits of CORM-3 on ischemic stroke through suppression of neuroinflammation and alleviation of BBB disruption.

## Methods

### Animals

We purchased adult, male C57BL/6 mice (12–14 weeks old, 25–30 g) from the Animal Experimental Center of Zhengzhou University. Efforts were made to minimize the number of animals used and their suffering.

### Transient middle cerebral artery occlusion model

We subjected mice to the tMCAO model of ischemic stroke as previously described [29–31]. We briefly anesthetized each mouse with an intraperitoneal injection of 5% chloral hydrate, made a midline neck incision, and carefully separated the arteries from adjacent tissues. Then, we introduced a 6.0 monofilament nylon suture with silicone-coated tip into the origin of the middle cerebral artery and left it in place for 60 min before withdrawing it. We defined a successful tMCAO as an approximately 80% decrease in cerebral blood flow confirmed by laser Doppler flowmetry (Moor Instruments, Devon, UK). Mice in the sham group were subjected to an identical surgical procedure, except that the filament was advanced to the origin of the middle cerebral artery and withdrawn immediately.

### Experimental groups

We randomly [32] assigned the animals to four groups: (1) sham mice treated with saline (Sham + saline, *n* = 72), (2) tMCAO-operated mice treated with saline (tMCAO + saline, *n* = 82), (3) sham mice treated with CORM-3 (Sham + CORM-3, *n* = 72), and (4) tMCAO-operated mice treated with CORM-3 (tMCAO + CORM-3, *n* = 82). We administered saline (vehicle) or CORM-3 (Sigma-Aldrich, St. Louis, MO, USA) dissolved in saline (4 mg/kg body weight) by retro-orbital injection at the time of reperfusion or 1 h after sham surgery [24, 33]. We examined mice for infection or illness daily.

### Cerebral infarct volume determination

We determined the brain infarct volume on days 1 and 3 after tMCAO as previously described [29, 34]. We sliced each brain into four, 2-mm-thick, coronal sections with a mouse brain matrix slicer (Stoelting Instruments, Wood Dale, IL, USA). The sections were immersed into 2% 2,3,5-triphenyltetrazolium chloride (TTC; Sigma-Aldrich) for 30 min at 37 °C and fixed in 4% paraformaldehyde in phosphate-buffered saline (PBS) overnight. Normal tissue stained red, whereas the infarct area remained white. We used Image J software (NIH, Bethesda, MD, USA) to measure the infarct area on the posterior surface in each brain slice by subtracting the intact area of the ipsilateral hemisphere from the total area of the contralateral hemisphere to correct for brain swelling. We then calculated the total infarct volume by linear integration of the corrected lesion areas [9]. We calculated the infarct volume percentage with the formula: total infarct volume/contralateral hemisphere volume × 100%.

### Evaluation of BBB permeability

For analysis of BBB permeability at 6 and 72 h post-surgery [9, 35], we injected each mouse with 100 μl of 4% Evans blue dye (dissolved in saline; Sigma-Aldrich) by retro-orbital injection. Two hours later, we perfused the mouse transcardially with saline, removed the brain, and separated it into ipsilateral and contralateral hemispheres. Each hemisphere was homogenized in *N*,*N*-dimethylformamide (Sigma-Aldrich) and centrifuged for 50 min at 14,000 rpm. We collected the supernatants and quantified Evans blue extravasation with the formula: (*A*_620 nm_ − ((*A*_500 nm_ + *A*_740 nm_)/2)) per gram wet weight. We subtracted background Evans blue level in the contralateral hemisphere from that of the ipsilateral hemisphere [8, 24]. For qualitative assessment of Evans blue extravasation at 72 h, we fixed the brains in 4% paraformaldehyde and cut 20-μm-thick sections. We mounted the sections on cover slips with a drop of mounting medium containing 1.5 μg/ml 4′,6-diamidino-2-phenylindole (DAPI; Santa Cruz Biotech, Dallas, TX, USA) and observed them under a fluorescence microscope (ZEISS Scope A1, ZEISS, Germany) [36].

### Assessment of brain water content

We sacrificed the mice on days 1 and 3 after tMCAO to determine the brain water content as previously described [30, 37]. The brain was divided into two hemispheres from the anatomic midline. The right hemisphere was immediately weighed with an electronic analytical balance to obtain the wet weight. The dry weights were obtained after the brain samples were dried at 100 °C in an oven for 24 h. We determined the brain water content with the following formula: (wet weight − dry weight)/wet weight × 100% [38].

### Neurologic assessment

An investigator blinded to the treatment groups tested the mice on days 1, 3, 7, and 14 after surgery for neurologic deficits using a five-point scale [8]: 0 = no neurologic deficit, 1 = failure to fully extend left forepaw, 2 = circling to the contralateral side, 3 = falling to the left, 4 = no spontaneous walking, 5 = depressed level of consciousness.

### Immunofluorescence

We sacrificed mice on day 3 after surgery for immunofluorescence analysis as previously described [30, 31, 39]. Briefly, mice were anesthetized with an intraperitoneal injection of 5% chloral hydrate and perfused with PBS and 4% paraformaldehyde. We carefully removed the brains, fixed them in 4% paraformaldehyde overnight at 4 °C, and then immersed them in 30% sucrose/PBS until they sank. Coronal sections (20 μm) were obtained by cryoultramicrotomy (CM1100, Leica Biosystems, Germany). After being washed in PBS, the sections were incubated in PBST (0.3% Triton X-100 in PBS), blocked in 1% bovine serum albumin (BSA)/PBST, and subsequently incubated with the following primary antibodies overnight at 4 °C: goat anti-Iba1 (1:1000, Abcam, Cambridge, MA, USA), rat anti-CD31 (1:500, Abcam), rabbit anti-ZO-1 (1:150, Proteintech, Sanying Biotechnology, Wuhan, China), rabbit anti-laminin (1:1000, Novus Biologicals, Littleton, CO, USA), rat anti-PDGFRβ (1:1000, Abcam), rabbit anti-MMP-9 (1:1000, Abcam). All antibodies were diluted in 1% BSA/PBST. Then, sections were washed in PBS and incubated with appropriate secondary antibodies for 1 h at room temperature in the dark. The sections were finally washed in PBS and mounted on cover slips with a drop of DAPI (Santa Cruz). Using a fluorescence microscope (ZEISS), an investigator blinded to the experimental groups randomly chose three separate tissue sections of each mouse and three non-overlapping 20× fields in the peri-infarction zone to quantify the number of activated microglia, the ZO-1-positive area/CD31-positive area, the laminin-positive area/CD31-positive area, and the number of PDGFRβ/MMP-9-positive cells. Activated microglia were defined as Iba1^+^ cells with a rod-like, spherical, or amoeboid appearance, and a cell body more than 10 μm in diameter that had short, thick processes and intense immunoreactivity, as we previously described [40, 41].

### Western blot analysis

We sacrificed the mice on day 3 after surgery for Western blot analysis as previously described [31, 40]. Protein was extracted from the whole right hemisphere, separated on 6–12% glycine gels, and transferred onto polyvinylidene difluoride membranes (Millipore, Bedford, MA). Membranes were blocked in 5% nonfat milk in Tris-buffered saline (TBS) with 0.1% Tween-20 (TBST) for 1 h at room temperature and stained overnight at 4 °C with one of the following primary antibodies: rabbit anti-NeuN (1:1000, Abcam), rabbit anti-MAP2 (1:200, Proteintech), rabbit anti-glyceraldehyde-3-phosphate dehydrogenase (GAPDH; 1:2000, Proteintech), goat anti-Iba-1 (1:1000, Abcam), rabbit anti-TNF-α (1:1000, Cell Signaling Technology, Danvers, MA, USA), rabbit anti-IL1β (1:200, Proteintech), rabbit anti-ZO-1 (1:200, Proteintech), rabbit anti-laminin (1:1000, Novus Biologicals), rat anti-PDGFRβ (1:1000, Abcam), rabbit anti-MMP-9 (1:50, Santa Cruz). These antibodies were diluted in 5% nonfat milk/TBST. We washed these membranes with TBST and incubated them with appropriate secondary antibodies conjugated to horseradish peroxidase for 2 h at room temperature. Protein bands were visualized by enhanced chemiluminescence detection kit (CW Biotech, Beijing, China), and an investigator blinded to the animal group quantified the optical density of the protein bands using Gel Analysis V 2.02 software (Clinx Science Instruments, Shanghai, China). GAPDH served as a loading control.

### Enzyme-linked immunosorbent assay (ELISA) analysis

We sacrificed the mice on day 3 post-surgery and prepared the brain homogenates for ELISA as previously described [31]. We homogenized the right hemisphere using a protein extraction kit (Sangon Biotech, Shanghai, China) and measured protein concentration with a bicinchoninic acid protein assay kit (Sangon Biotech). We adjusted the total protein concentration to 1 mg/ml protein extract and quantified the concentrations of TNF-α and IL1β in brain homogenates according to the manufacturer’s protocol (Boster, Wuhan, China).

### Statistical analysis

We performed statistical analysis with SPSS version 13.0. Results are expressed as mean ± SD. We used repeated measures ANOVA to determine changes in neurologic deficit score after tMCAO. Student’s *t* test or one-way ANOVA followed by the least significant difference test was used to analyze differences in data from infarction volume, brain water content, Evans blue dye leakage, Western blot analysis, ELISA analysis, and immunofluorescence. Differences were considered statistically significant at *p* < 0.05.

## Results

### CORM-3 treatment reduces cerebral infarct volume and increases protein levels of NeuN and MAP2 after tMCAO

**Fig. 1:**
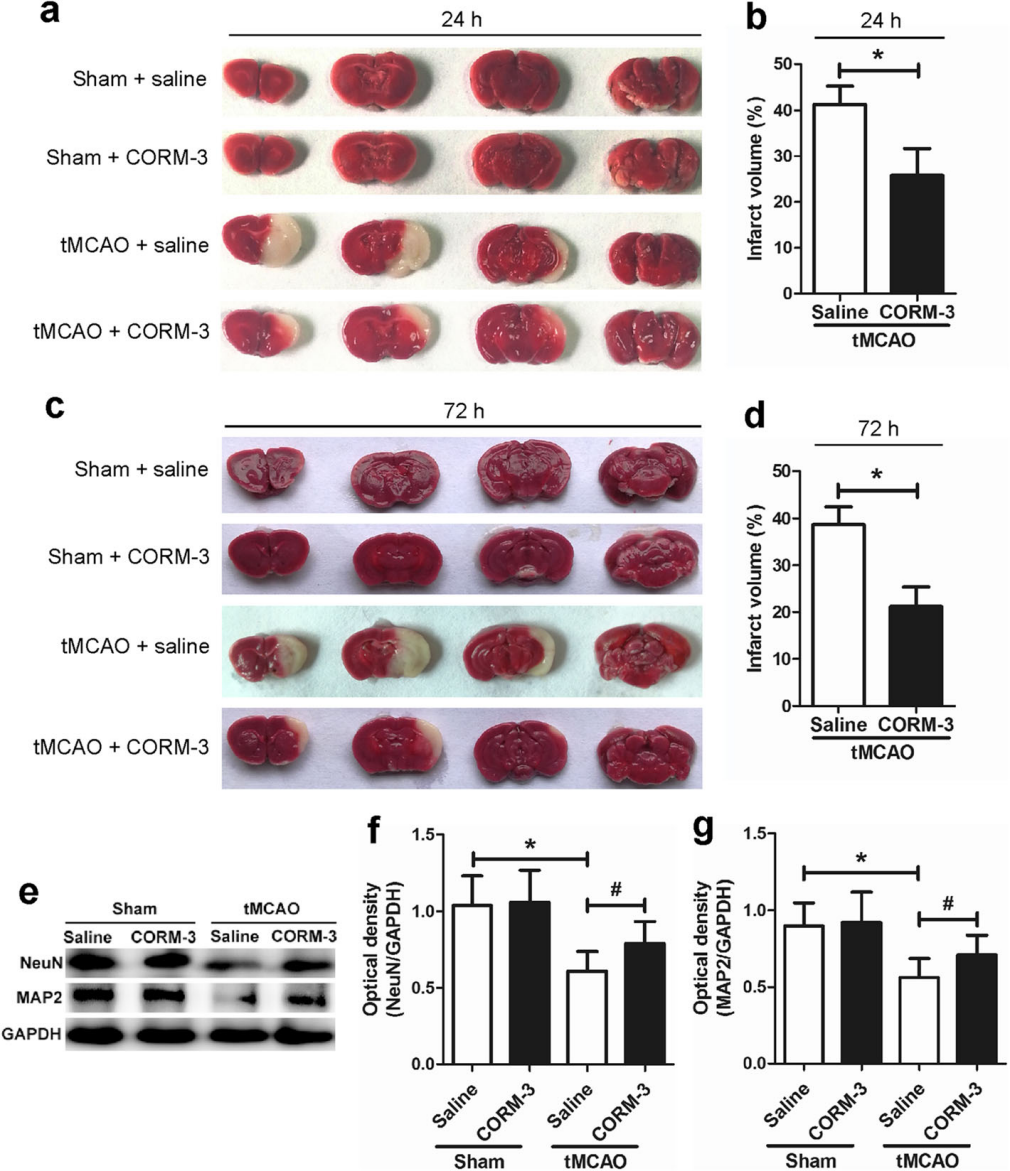
CORM-3 treatment reduces infarct volume and increases the expression of NeuN and MAP2. **a**, **c** Representative TTC staining illustrates the brain infarct volume in each group at 24 h (**a**) and 72 h (**c**) after tMCAO or sham surgery; areas of infarct remain white. **b**, **d** Quantification of TTC staining in the saline- and CORM-3-treated tMCAO groups at 24 h (**b**) and 72 h (**d**) after tMCAO. Values represent corrected infarct volume as a percentage of the contralateral area (*p < 0.05, *n* = 6 per group). **e** Representative Western blot analysis of NeuN and MAP2 in the right hemisphere of each group. **f**, **g** Quantification of the Western blot analysis (*p < 0.05 vs. Sham + saline group, ^#^*p* < 0.05 vs. tMCAO + CORM-3 group, *n* = 10 per group). Data are shown as mean ± SD

TTC staining showed that the infarct volume of the tMCAO + saline group was significantly larger than that of the tMCAO + CORM-3 group on days 1 and 3 after surgery (Fig. 1a–d). Western blot analysis revealed that the expression levels of NeuN and MAP2 were significantly lower in the tMCAO + saline group than in the Sham + saline group on day 3. The CORM-3-treated tMCAO group had significantly higher levels of NeuN and MAP2 than did the saline-treated tMCAO group on day 3 after brain ischemia. Additionally, the protein levels of NeuN and MAP2 did not differ significantly between the Sham + saline group and the Sham + CORM-3 group (Fig. 1e–g).

### CORM-3 treatment decreases the number of activated microglia and the expression of Iba-1, TNF-α, and IL1β after tMCAO

**Fig. 2:**
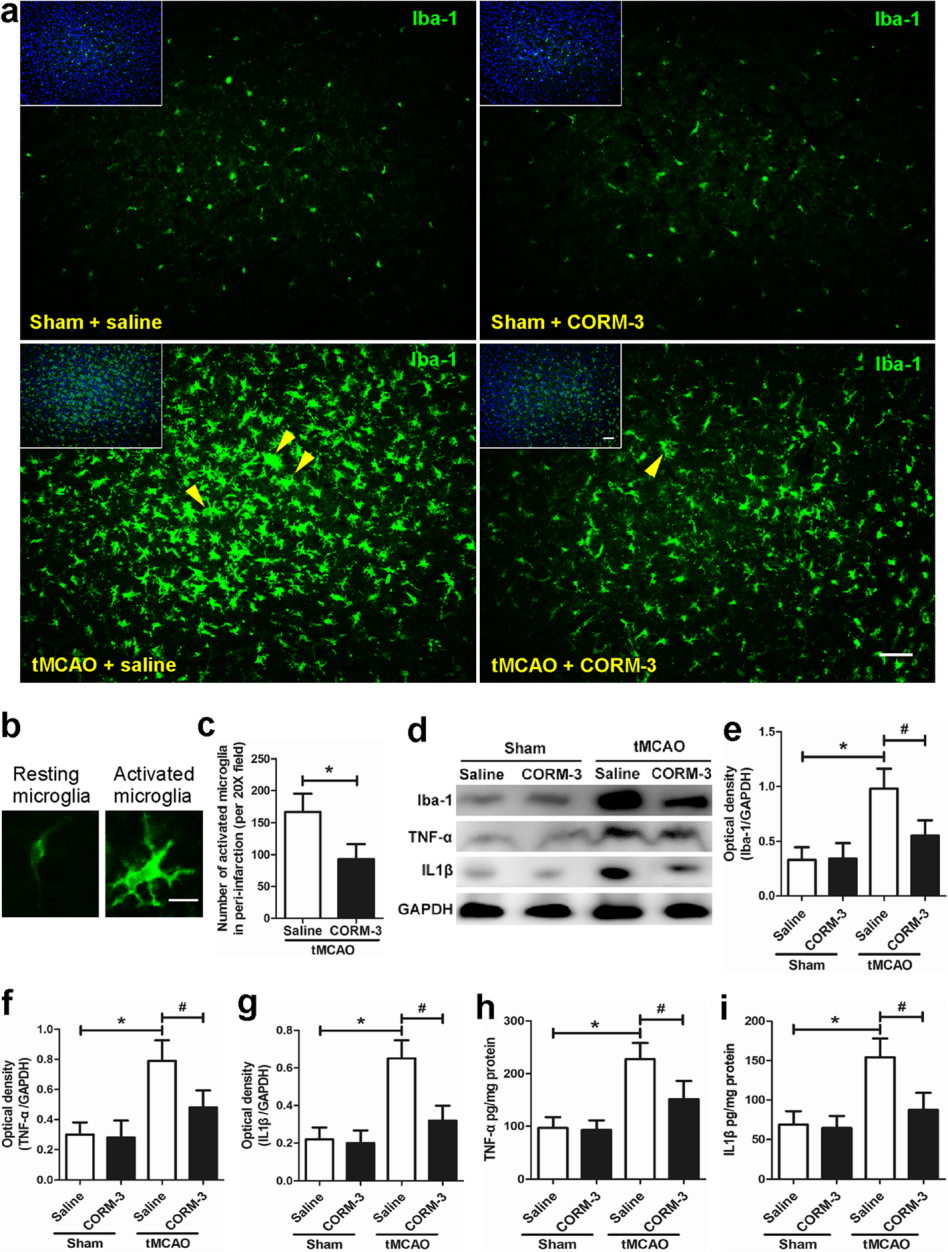
CORM-3 treatment decreased the number of activated microglia at 3 days after ischemia. **a** Representative immunofluorescence staining of Iba-1-positive cells in each group. Insets show colocalization of the nuclear stain DAPI (blue). Yellow arrowheads point out activated microglia in peri-infarction zones. Scale bar = 50 μm. **b** Representative images show the morphology of resting (left) and activated (right) microglia. Scale bar = 10 μm. **c** Quantification indicates that CORM-3 significantly decreased the number of activated microglia (*p < 0.05, *n* = 8 per group). **d** Representative Western blot of Iba-1, TNF-α, and IL1β expression. **e**–**g** Quantification of the Western blot analysis (*p < 0.05 vs. Sham + saline group, ^#^*p* < 0.05 vs. tMCAO + CORM-3 group, *n* = 10 per group). **h**, **i** ELISA analysis of TNF-α and IL1β (*p < 0.05 vs. Sham + saline group, ^#^*p* < 0.05 vs. tMCAO + CORM-3 group, *n* = 8 per group). Data are shown as mean ± SD

In comparison with saline-treated tMCAO mice, CORM-3-treated tMCAO mice had significantly fewer activated microglia in the peri-infarction zone on day 3 after ischemia (Fig. 2a–c), a finding that was confirmed by the concomitant reduction in Iba-1 protein expression (Fig. 2d, e). Expression levels of pro-inflammatory cytokines TNF-α and IL1β were significantly higher in the tMCAO + saline group than that in the Sham + saline group, but CORM-3 significantly prevented this increase, as evidenced by Western blot results on day 3 after surgery. Levels of both TNF-α and IL1β were similar between the saline-treated and CORM-3-treated sham groups (Fig. 2d, f–i).

CORM-3 treatment reduces brain water content and neurologic deficits after tMCAO.

**Fig. 3:**
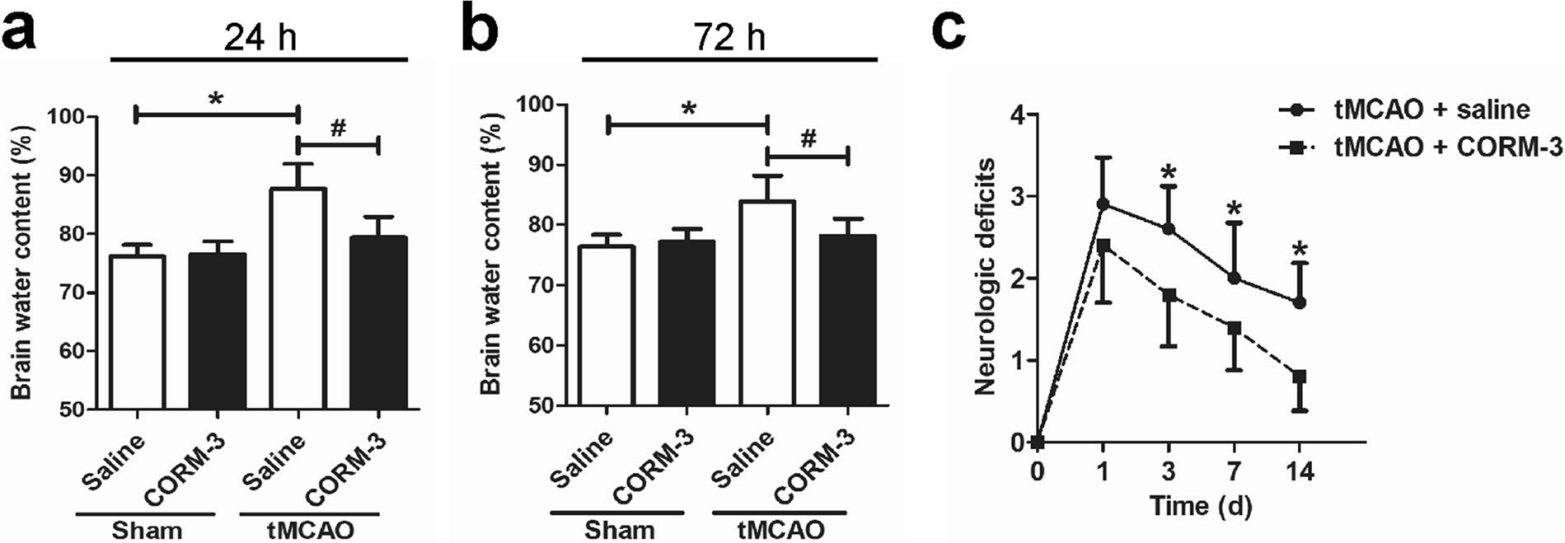
Assessments of brain water content and neurologic deficit. **a**, **b** Quantification of brain water content in the indicated groups at 24 h (**a**) and 72 h (**b**) after tMCAO or sham surgery (*p < 0.05 vs. Sham + saline group, ^#^*p* < 0.05 vs. tMCAO + CORM-3 group, *n* = 6 per group). **c** Neurologic deficits were significantly less in the CORM-3-treated tMCAO mice than in the saline-treated tMCAO mice on days 3, 7, and14 (*p < 0.05, *n* = 10 per group). Data are shown as mean ± SD

On days 1 and 3 after the ischemic insult, we found that saline-treated tMCAO mice had higher brain water content than the saline-treated sham mice; however, brain water content was significantly lower in the tMCAO + CORM-3 group than in the tMCAO + saline group. No significant difference was apparent between the Sham + saline and the Sham + CORM-3 groups (Fig. 3a, b). Neurologic deficit scores were elevated after tMCAO and gradually decreased over 2 weeks. Notably, tMCAO mice that received CORM-3 had lower scores on days 3, 7, and 14 than did the saline-treated tMCAO mice (Fig. 3c).

### CORM-3 treatment reduces Evans blue leakage after tMCAO

**Fig. 4:**
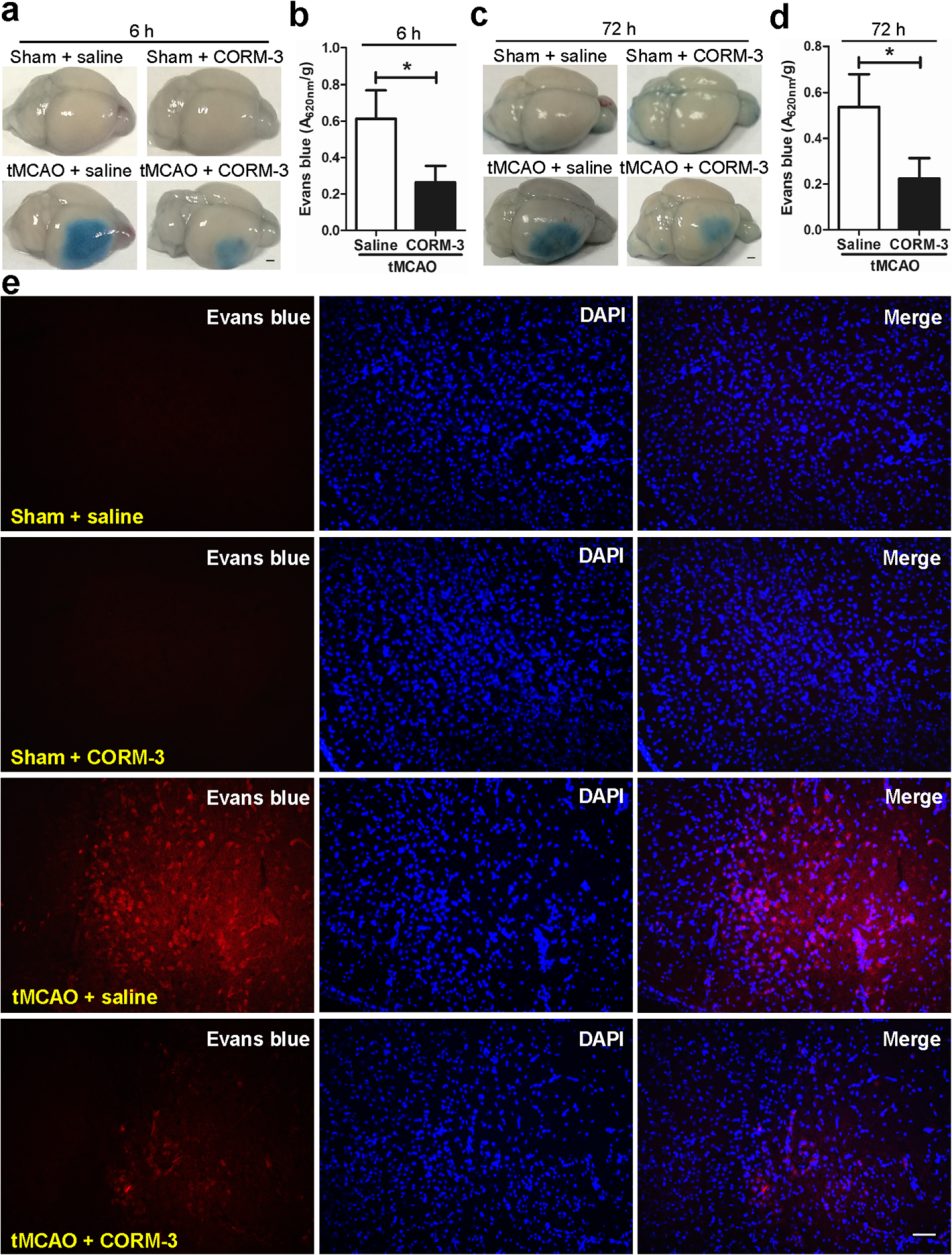
Assessment of blood-brain barrier dysfunction. **a**, **c** Representative brain images show Evans blue leakage at 6 h (**a**) and 72 h (**c**) post-surgery in each group. Scale bar = 1 mm. **b**, **d** Quantitative analysis of Evans blue leakage in tMCAO + saline and tMCAO + CORM-3 groups at 6 h (**b**) and 72 h (**d**) post-surgery (*p < 0.05, *n* = 8 per group, data are shown as mean ± SD). **e** Representative fluorescence of Evans blue extravasation in peri-infarction zone at 72 h post-surgery (*n* = 6 per group). Scale bar = 50 μm

We evaluated BBB permeability at 6 and 72 h after surgery by assessing Evans blue extravasation. The results showed that the Evans blue leakage was significantly reduced in the tMCAO + CORM-3 group compared to that in the tMCAO + saline group (Fig. 4a–d). The qualitative assessment of Evans blue extravasation at 72 h further confirmed the findings (Fig. 4e).

### CORM-3 treatment increases the expression of ZO-1 and laminin after tMCAO

**Fig. 5:**
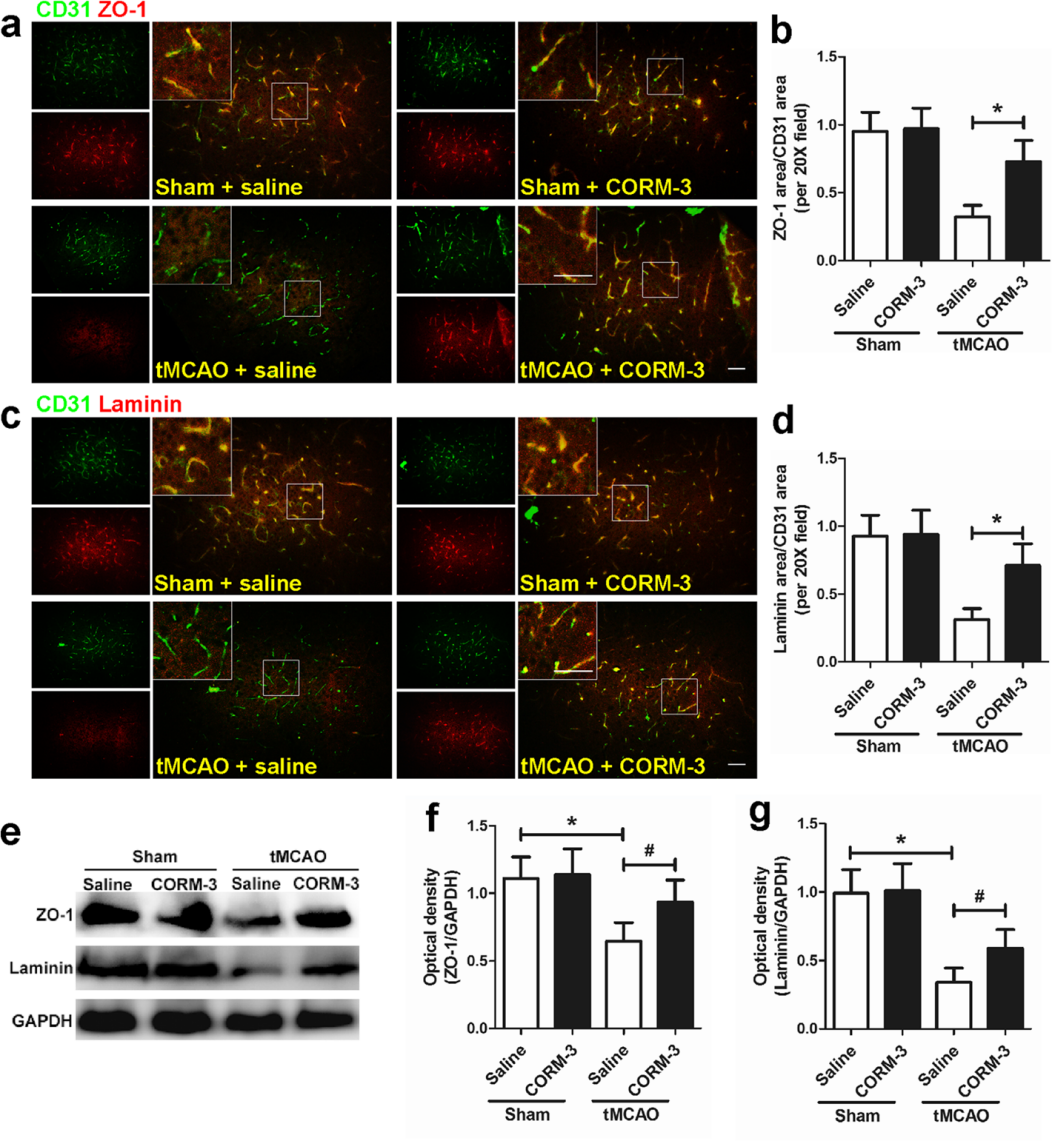
CORM-3 increases the expression of ZO-1 and laminin on day 3 after cerebral infarction. **a** Representative images show double immunofluorescence staining of CD31/ZO-1-positive cells in peri-infarction zones. Insets show high-magnification images of the boxed areas. Scale bar = 50 μm. **b** Quantification of ZO-1-positive area/CD31-positive area in peri-infarction zones (*p < 0.05, *n* = 8 per group). **c** Representative images show double immunofluorescence staining of CD31/laminin-positive cells in peri-infarction zones. Insets show high-magnification images of the boxed areas. Scale bar = 50 μm. **d** Quantification of laminin-positive area/CD31-positive area in peri-infarction zones (*p < 0.05, *n* = 8 per group). **e** Representative Western blot of ZO-1 and laminin expression in the right hemisphere of each group. **f**, **g** Quantification of the Western blot analysis (*p < 0.05 vs. Sham + saline group, ^#^*p* < 0.05 vs. tMCAO + CORM-3 group, *n* = 10 per group). Data are shown as mean ± SD

Immunofluorescence staining showed that CORM-3-treated tMCAO mice had more ZO-1-positive or laminin-positive vessels (labeled by CD31) in peri-infarction regions than did saline-treated tMCAO mice on day 3 post-surgery (Fig. 5a–d). Western blot analysis showed that expression levels of ZO-1 and laminin were significantly higher in the CORM-3-treated tMCAO mice than in the saline-treated tMCAO mice. However, the expression of neither protein differed significantly between the saline-treated and CORM-treated sham mice (Fig. 5e–g).

### CORM-3 treatment increases the expression of PDGFRβ and decreases the expression of MMP-9 after tMCAO

**Fig. 6:**
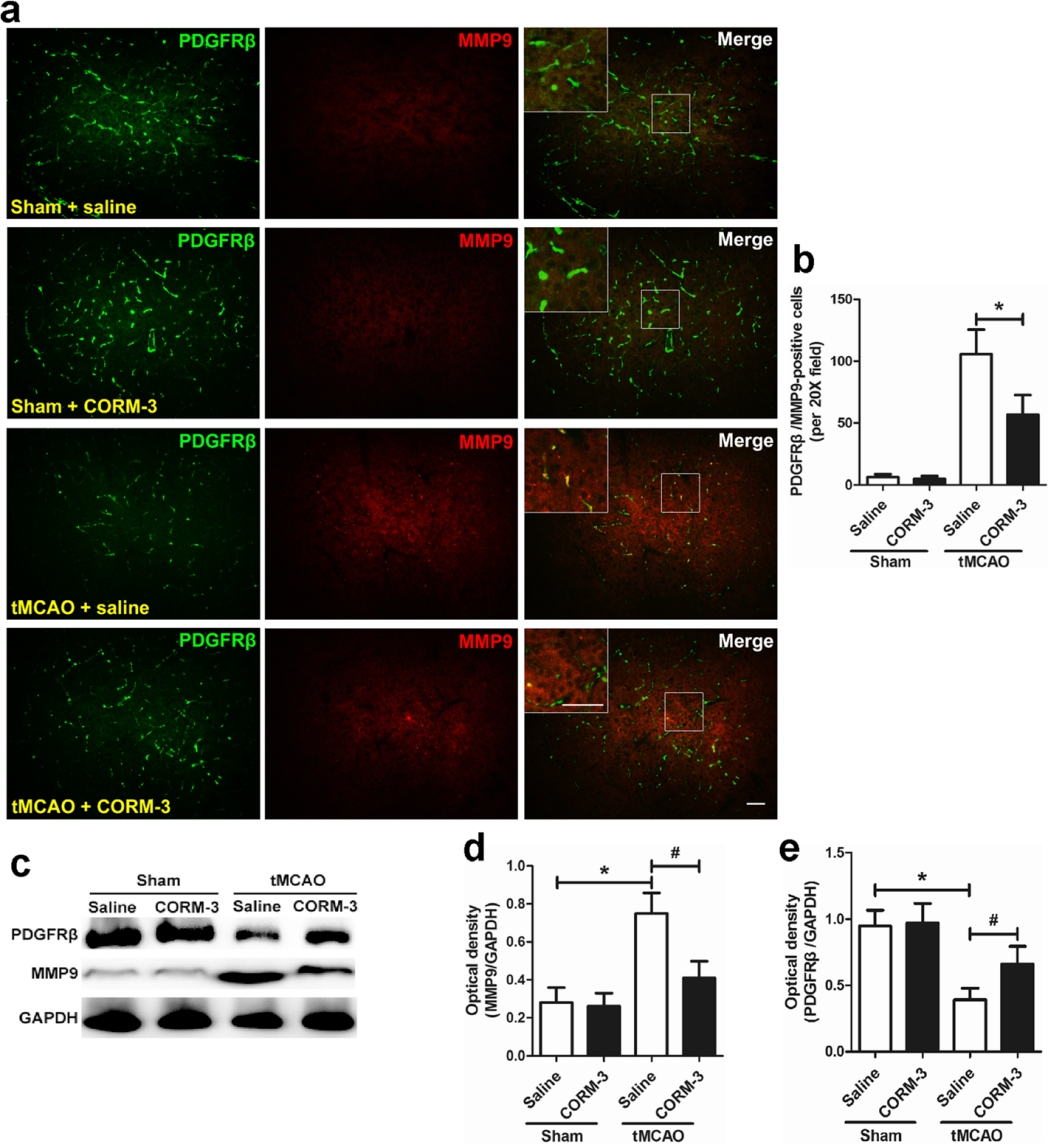
Effects of CORM-3 on the expression of PDGFRβ and MMP-9 on day 3 after ischemic stroke. **a** Representative images show double immunofluorescence staining of PDGFRβ/MMP-9-positive cells in peri-infarction zones. Insets show high-magnification images of the boxed areas. Scale bar = 50 μm. **b** Quantification of the double immunofluorescence staining (*p < 0.05, *n* = 8 per group). **c** Representative Western blot of PDGFRβ and MMP-9 expression in the ipsilateral tissue of each group. **d**, **e** Quantification of the Western blot analysis (*p < 0.05 vs. Sham + saline group, ^#^*p* < 0.05 vs. tMCAO + CORM-3 group, *n* = 10 per group). Data are shown as mean ± SD

We detected the expression of PDGFRβ and MMP-9 in the ischemic hemisphere by using immunofluorescence staining and Western blot analysis on day 3 after surgery. The tMCAO + CORM-3 group had significantly fewer PDGFRβ/MMP-9-positive cells in peri-infarction areas than did the tMCAO + saline group (Fig. 6a, b). The tMCAO + saline group had significantly lower expression of pericyte marker PDGFRβ and greater expression of MMP-9 than did the Sham + saline group. On the other hand, the tMCAO + CORM-3 group had significantly higher expression of PDGFRβ and lower expression of MMP-9 when compared with levels in the tMCAO + saline group. There were no differences in the protein levels between Sham + saline and Sham + CORM-3 groups (Fig. 6c–e).

## Discussion

Our findings suggest that CORM-3 has a protective effect in a mouse model of ischemic stroke injury. We found that CORM-3 treatment reduced infarct volume and brain water content, alleviated BBB disruption, and promoted neurologic recovery after ischemic stroke. Furthermore, CORM-3 increased the protein levels of NeuN and MAP2, reduced microglial activation, decreased the expression of pro-inflammatory cytokines TNF-α and IL1β, increased the expression of PDGFRβ, ZO-1, and laminin, and downregulated the protein level of MMP-9 compared with corresponding levels in saline-treated mice. Our study suggests that CORM-3 ameliorates ischemic stroke injury by suppressing neuroinflammation and alleviating BBB disruption.

Against general dogma that CO is poisonous, particularly to the brain, accumulating evidence suggests that it might be associated with cytoprotection and maintenance of homeostasis in several organs and tissues [10, 23, 27, 28, 42–44]. Therefore, exogenous administration of CO at low levels (as inhaled CO gas or as CORMs) could be explored as a potential therapeutic method. It has been shown that low levels of inhaled CO provide benefit against ischemia-reperfusion brain injury by attenuating ischemia-induced infarct volume, reducing edema formation, and restoring cerebral blood flow [45]. A study that used a model of permanent ischemic stroke indicated that a low concentration of inhaled CO provided neuroprotective effect by activating the Nrf2 pathway [46]. However, the application of CO gas presents several limitations, such as the potential for partial systemic hypoxia and toxicity and the need for CO inhalation facilities and monitoring of blood oxygen levels [11]. Given these barriers, transition metal carbonyls are good candidates for delivering CO, as they function as CORMs in biological systems [11]. Water-soluble CORM-3 has beneficial effects in many models of injury, such as hemorrhagic stroke, pulmonary hypertension, and ischemia-reperfusion injury during kidney transplantation [21, 22, 47]. However, the effect of CORM-3 on ischemic stroke and its mechanism of protection are still unclear. In this study, we treated tMCAO mice with CORM-3 at the time of reperfusion and investigated the therapeutic effects. We used a CORM-3 dose of 4 mg/kg because of studies showing that this dose releases an amount of CO in mice that may be sufficient to exert protective effect [22, 24]. We did not monitor the effects of CORM-3 on the level of COHb because studies have already indicated that various doses of CORM-3 have no effects on COHb in mice [19, 20]. We found that the administration of CORM-3 significantly decreased cerebral infarct volume and water content after ischemic stroke, similar to results from a previous study that used inhaled CO [45]. Additionally, the protein levels of NeuN and MAP2 in the ischemic brain of CORM-3-treated tMCAO mice were significantly increased compared with those of saline-treated controls. NeuN and MAP2 are well recognized as markers of mature neurons. It is likely that CORM-3 increases the levels of these two proteins by decreasing neuronal death or apoptosis, rather promoting neurogenesis, because 3 days is not long enough for neural stem cells in the subventricular zone to proliferate, migrate to the peri-infarction zone, and differentiate into mature neurons [40, 48, 49]. In addition, CORM-3 treatment markedly reduced BBB permeability and promoted neurologic recovery after ischemic stroke. These results suggest that CORM-3 might provide protection against ischemic stroke. Our study focused on the acute phase after ischemia, but it will be important to determine the long-term effects of CORM-3 on post-stroke neurogenesis, angiogenesis, and recovery of ischemic white matter injury.

Upregulation of endogenous neuroinflammatory processes is a crucial secondary cell death mechanism that follows ischemic stroke and initiates a feedback loop of inflammatory cascades that can expand the region of brain damage [1]. Evidence has shown that inflammation not only affects the infarct tissue in the near-term but also causes long-term damage in the ischemic penumbra [2, 4]. Microglia, the brain’s resident immune cells, are rapidly activated from their resting state after ischemic stroke and are considered to be major cellular contributors to neuroinflammation [50, 51]. Activated microglia initiate neuroinflammation by producing cytotoxic and inflammatory factors, including the cytokines TNF-α and IL-1β, thereby aggravating brain damage [5, 52]. At high concentrations, TNF-α and IL1β have direct toxic effects on neurons and neural cells [53, 54]. Consequently, activated microglia have been the target of studies focusing on neuroinflammation. CORMs possess anti-inflammatory properties, and CORM-3 has been shown to reduce microglial activation in BV-2 microglia and in a rat model of hemorrhagic stroke [22, 55]. To investigate whether CORM-3 regulates neuroinflammation after ischemic stroke, we characterized activated microglia as Iba1^+^ cells with special morphology as previously described [14, 40, 56]. We found that the administration of CORM-3 decreased the number of activated microglia in the peri-infarction zone and reduced the expression of Iba-1, TNF-α, and IL1β in ipsilateral tissue at 72 h after reperfusion. Consistent with the anti-inflammatory effect shown in previous studies, our results indicate that CORM-3 suppressed neuroinflammation after ischemic stroke. We hypothesize that CORM-3 might reduce the release of damage-associated molecular pattern molecules (DAMPs), such as high-mobility group box 1 (HMGB1) and galectin-3 (Gal3) [57], which have been shown to stimulate microglia after ischemic stroke. HMGB1 is released from necrotic neurons and mediates the activation of microglia via Toll-like receptors (TLRs) or the receptor of advanced glycation end-products (RAGE) on microglia [58]. Gal3 is released from microglia, and Gal3-dependent TLR4 activation promotes microglial activation [59]. CORM-3 might directly modulate microglia phenotype by inhibiting microglial nucleotide-binding domain (NOD)-like receptor protein 3 (NLRP3) signaling, but this possibility needs to be further investigated. In addition to the resident microglial activation, the infiltration of circulating monocytes/macrophages is considered to play a vital role in post-stroke neuroinflammation [57]. Details regarding the mechanisms that underlie the effects of CORM-3 on microglia and macrophages after ischemic stroke require additional exploration.

Normally, the BBB protects the brain from toxic substances. BBB disruption is a crucial event in the pathogenesis of acute ischemic stroke that leads to cerebral edema, brain hemorrhage, and neuronal death [7]. Previous data suggest that overexpression of metalloproteinases, especially MMP-9, after ischemic stroke disrupts the BBB by degrading the tight junctions and basal lamina proteins [24, 60–63]. Furthermore, a recent study showed that pericytes, a key component of the BBB, contribute to BBB disruption in the capillary bed during acute ischemic stroke, with rapid MMP-9 activation at pericyte somata before capillary leakage [64]. In a mouse model of traumatic brain injury, CORM-3 was shown to reduce pericyte death and MMP-9 expression at pericyte somata [24]. However, the effect of CORM-3 on BBB disruption after ischemic stroke remains unknown. In this study, we showed that CORM-3 treatment reduced Evans blue leakage and brain edema, indicating that it can reduce BBB permeability in mice after tMCAO. The administration of CORM-3 increased the expression of PDGFRβ, ZO-1, and laminin and decreased the expression of MMP-9, indicating that it can protect pericytes, tight junction proteins, and matrix proteins after tMCAO. These results suggest that CORM-3 can potentially protect BBB integrity from ischemic stroke injury. Although we showed that CORM-3 decreased MMP-9 expression after ischemia, its effect on MMP-9 function would be better supported by gelatin gel zymography assessment [65]. Considering the peak concentration of reactive oxygen species (ROS) during reperfusion [66], we hypothesize that CORM-3 may rescue pericytes after tMCAO by stabilizing the ROS/hypoxia-inducible factor-1α axis, leading to microvascular stabilization. Although studies have shown that CORM-3-derived CO may be a potent enhancer of angiogenesis in vitro [67], and pericytes may contribute to post-stroke angiogenesis [68–70], it would be interesting to identify the direct effect of CORM-3 on endothelial cells after ischemic stroke, and the indirect effect of CORM-3 on angiogenesis, for example whether CORM-3 affects pericyte-endothelial cell crosstalk after stroke.

One limitation of our study is that we administered CORM-3 only at the time of reperfusion by way of retro-orbital injection. The effects of CORM-3 administrations at different time points and by different routes should be further investigated. Additionally, microscopic analysis of the brains was made only on day 3 after tMCAO, which may be sufficient to support our conclusion, but longitudinal observations, such as on days 7, 14, and 21, are needed to clarify the mechanism by which CORM-3 promotes post-stroke recovery. It would be interesting to study the mechanism by which CORM-3 treatment suppresses microglial activation in the ischemic brain and to determine whether CORM-3 affects newly generated microvessels. Identification of the long-term effects of CORM-3 on ischemic stroke will provide better support for the potential therapeutic value of the CORMs.

## Conclusions

In conclusion, we showed that CORM-3 treatment at the time of reperfusion exerts beneficial effects on ischemia-reperfusion brain injury by suppressing neuroinflammation and alleviating BBB disruption. Our findings support the premise that CORM-derived CO may potentially be applied in the treatment of ischemic stroke.

## Abbreviations

BBB: Blood-brain barrier
BSA: Bovine serum albumin
CO: Carbon monoxide
COHb: Carboxyhemoglobin
CORM-3: Carbon monoxide-releasing molecule-3
DAPI: 4′, 6-Diamidino-2-phenylindole
Iba-1: Ionized calcium-binding adapter molecule-1
IL-1β: Interleukin 1β
MAP 2: Microtubule-associated protein 2
MMP-9: Matrix metalloproteinase-9
NeuN: Neuronal nuclear antigen
PBS: Phosphate-buffered saline
PDGFR-β: Platelet-derived growth factor receptor-β
TBS: Tris-buffered saline
tMCAO: Transient middle cerebral artery occlusion
TNF-α: Tumor necrosis factor-α
TTC: 2,3,5-Triphenyltetrazolium chloride
